# Ergothioneine supplementation improves pup phenotype and survival in a murine model of spinal muscular atrophy

**DOI:** 10.1002/1873-3468.70136

**Published:** 2025-08-06

**Authors:** Francesca Cadile, Daniela Ratto, Giorgia Rastelli, Ottavia Eleonora Ferraro, Caterina Temporini, Sunil Kumar, Simona Boncompagni, Paola Rossi, Monica Canepari

**Affiliations:** ^1^ Department of Molecular Medicine, Via Forlanini 6 University of Pavia Italy; ^2^ Department of Biology and Biotechnology “L. Spallanzani” University of Pavia Italy; ^3^ CAST, Center for Advanced Studies and Technology University G. D'Annunzio of Chieti‐Pescara Italy; ^4^ Department of Public Health, Experimental and Forensic Medicine, Unit of Biostatistics and Clinical Epidemiology University of Pavia Italy; ^5^ Department of Drug Sciences, Via Taramelli 12 University of Pavia Italy; ^6^ DNICS, Department of Neuroscience, Imaging and Clinical Sciences University G. D'Annunzio of Chieti‐Pescara Italy

**Keywords:** antioxidant, diaphragm muscle, ergothioneine, mitophagy, spinal muscular atrophy

## Abstract

Spinal muscular atrophy (SMA) is a genetic disorder characterized by the loss of spinal motor neurons. The conventional therapy does not always lead to a full restoration of the clinical symptoms, partially due to the need for early treatment. Accumulating evidence describes the crucial role of mitochondrial dysfunction and oxidative stress in skeletal muscle of SMA patients. We aimed to investigate the effects of prenatal supplementation with the antioxidant molecule ergothioneine (ERGO) on an SMNΔ7 mouse model of SMA containing a knockout of survival motor neuron protein (SMN1) and two transgenes, one with a single normal copy of human SMN2 and the second with a human SMN2 promoter and a human SMN2 cDNA lacking exon 7. ERGO had a significant positive effect on the survival and locomotor abilities of SMA pups. In isolated diaphragm muscle, ERGO was found to stimulate mitophagy. The results of the current study highlight the need for further research into ERGO as an adjuvant therapy for SMA.

**Impact statement**

Our finding that ergothioneine supplementation improves survival in a murine model of spinal muscular atrophy may aid research into a novel potential adjuvant to alleviate the symptoms of this serious neuromuscular disease in humans.

## Graphical abstract

Spinal muscular atrophy (SMA) is a genetic disease affecting motor neurons. Individuals with SMA experience mitochondrial dysfunction and oxidative stress. The aim of the study was to investigate the effect of an antioxidant and neuroprotective substance, ergothioneine (ERGO), on an SMNΔ7 mouse model of SMA. The treatment of pregnant mice with ERGO improved the survival and motor function of SMA pups.

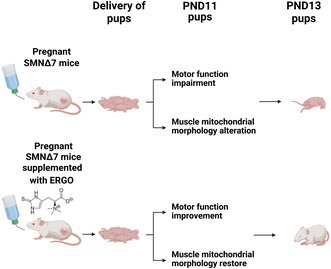

## Abbreviations

**APS**, Aggregate Phenotypic Score

**CRUs**, Ca2+ release units

**EM**, electron microscopy

**ERGO**, ergothioneine

**HLS**, hind limb score

**MNs**, spinal motor neurons

**OI**, oxidative index

**ROS**, reactive oxygen species

**SMA**, spinal muscular atrophy

**SMN**, survival motor neuron

Spinal muscular atrophy (SMA) is a genetic disorder characterized by the loss of spinal motor neurons (MNs). The disease is caused by mutations in the *SMN1* gene, which encodes the survival motor neuron (SMN) protein. The low expression of SMN protein is the main causative factor of SMA; however, increased findings demonstrate the presence of mitochondrial dysfunction and oxidative stress in tissue and cells relevant to SMA pathology [1]. Accumulation of reactive oxygen species (ROS) leads to MNs death as well as tissue damage in various neurodegenerative disorders [2, 3]. Unsurprisingly, energetic disturbances suggestive of underlying mitochondrial dysfunction are present in muscle biopsies from patients and mouse models of SMA [4, 5, 6]. The precise role or roles of *SMN1* in mitochondrial health and homeostasis remain unclear. However, ROS can reduce SMN levels, inducing disulfide crosslinking and inactivation of the protein, exacerbating the SMA phenotype [7].

Significant progress has been made recently for SMA treatment with the approval of three drugs, Spinraza (Nursinersen), Risdiplam (Evrysdi), and Onasemnogene Abeparvovec (Zolgensma), that are able to increase the level of SMN protein. Current therapies have made impressive strides in extending patients life expectancy and improving their motor function. However, for many patients, the burden of SMA remains challenging due to ongoing muscle weakness and other skeletal problems. It has been demonstrated by a transcriptional analysis the persistence of mitochondrial alterations in the muscles of patients treated with Nusinersen or Risdiplam [8], supporting the need for combination therapies targeting the OXPHOS pathway. Another important point to be considered is the need for early treatment. Since much of MNs development and systemic pathomechanistic changes are occurring prenatally, a prenatal therapy can have added benefit in order to rescue some irreversible damage [9, 10]. Regarding this aspect, on 19 February 2025, a letter was published reporting the first single case of prenatal therapy with Risdiplam [11]. In this study, a 2‐year‐old girl was successfully treated for SMA while still in the womb taken by her mother during pregnancy, and the child currently shows no signs of the disease.

For the reasons cited above, it is critical that we continue to learn about every facet of SMA to improve existing therapies and create combinatorial ones [9, 12, 13]. A treatment targeting mitochondria and/or ROS could exert a positive effect on the overall outcome of this neuromuscular disease. Similar strategies stimulating mitochondrial biogenesis have proven useful in contrasting neurological disorders [14, 15, 16, 17].

The SMA treatment with the antioxidant Olesoxime (a mitochondria‐targeting cholesterol derivative) has emerged as a promising drug. Regretfully, Roche declared in 2018 that the phase 3 clinical trial would be discontinued because of issues with dose and production, in addition to unsatisfactory long‐term outcomes [18].

Ergothineine (ERGO) is a water‐soluble natural antioxidant able to permeate the placenta, brain barrier, and mitochondrial membranes, to scavenge mitochondria‐derived ROS and protect mitochondrial constituents from damage by ROS [19, 20, 21]. ERGO has been demonstrated to attenuate oxidative stress and nitrosamine damage in injured or aging brain [22]. It has been demonstrated that standardized extracts of medicinal mushrooms containing a known quantity of ERGO improve recognition memory and locomotor performances both in wild‐type (WT) animals [20, 21] and in frail elderly mice [23, 24, 25]. Furthermore, ERGO is able to reduce exercise‐related stress after acute exercise in adult healthy mice [26] and can counteract fatigue in isolated diaphragm of the SMNΔ7 mice [6]. A study of toxicology giving enormous doses of ERGO to pregnant rats demonstrates the reproductive safety of ERGO [27] and based on this study the European Food Safety Authority (EFSA) has granted use for ERGO as a food additive in pregnant women and infants [28]. Moreover, ERGO can rescue fetal growth restriction in the preclinical model of preeclampsia [29]. ERGO is thought to be an adaptive antioxidant, that is, its properties come into play mainly when the tissues are damaged by increasing the expression of ERGO transporter OCTN1 [30, 31, 32].

Its multiple properties and its high bioavailability make it an excellent potential dietary supplement for SMA patients. The current study aimed to understand whether supplementation, during gestation and lactation with ERGO, could ameliorate the phenotype of SMNΔ7 mouse model of SMA.

## Materials and methods

### Animals and treatment

Mice showing SMA phenotype and control littermate were used (SMNΔ7 mice from Jackson Laboratory stock #005025). The animals will be monitored several times a day in order to evaluate the onset of severe motor and/or respiratory disorders, food and/or water refusal. Since the SMNΔ7 mouse model develops a symptomatology similar to human SMA for several aspects, careful monitoring of the animals' clinical manifestations will be used in order to allow an appropriate intervention aimed at partially alleviating the suffering of the animals with motor deficits.

This mouse model is a triple mutant that contains a knockout of mouse Smn1 (caused by insertion of a lacZ reporter) and two transgenes, one with a single normal copy of human SMN2 and the second with a human SMN2 promoter and a human SMN2 cDNA that lacks exon 7.

Mating a heterozygous male with a heterozygous female, we obtained:

1. Homozygous mice for both transgenes and heterozygous mice for the null allele (7SMN^+/+^; SMN2^+/+^; SMN^+/−^—50%). These mice were used as breeders.
2. Homozygous mice for all three alleles (7SMN^+/+^; SMN2^+/+^; SMN^+/+^—25%). These mice were named WT.
3. Homozygous mice for both transgenes and homozygous mice for the null allele (7SMN^+/+^; SMN2^+/+^; SMN^−/−^—25%). These mice were named SMA.

To evaluate the effect of ERGO, a dietary supplementation was carried out in heterozygous SMNΔ7 pregnant/maternal mice by adding ERGO to the drinking water during pregnancy and lactation time. We measured the average daily water intake of the pregnant and lactating mice and adjusted the concentration of ERGO in their drinking water to ensure a daily intake of 20 μg. The dose of 20 μg/day was chosen based on a balance of safety, effectiveness, and physiological relevance to maximize beneficial effects on newborn mice during critical developmental periods [24, 25]. Our aim was to model a nutritional or dietary supplementation scenario consistent with potential human consumption levels [25] For a control measurement, we also recorded the water consumption of a single mouse. Pure ERGO was provided by Tetrahedron (Paris, France). ERGO can cross the blood–brain barrier and the placental barrier and is present in breast milk [33].

The pups from the treated mother will be WT + ERGO and SMA + ERGO. They will continue to take ERGO during breastfeeding through the mother's milk until the eleventh day of life.

All experimental procedures will be performed on 11‐day‐old male and female mice.

Mice were genotyped utilizing a PCR assay and euthanized using cervical dislocation. The lower part of the thorax was excised, and the diaphragm muscle was dissected.

All the procedures were approved by the Animal Care and Use Committee at the University of Pavia (protocol reference number: 280/2021‐PR) and were communicated to the Ministry of Health and local authorities by Italian law.

### Genotyping

A PCR‐based assay on tail DNA was used to genotype offspring. The mouse SMN knockout allele was detected using the primers (Sigma Aldrich, St. Louis, MO, USA) reported in Table 1. The PCR procedure was described in Cadile *et al*. [6].

**Table 1:** Primers used for PCR assay to genotype offspring.

Gene	Primers
SMN1 WT FORWARD	5′ CTCCGGGATATTGGGATTG 3′
SMN1 WT REVERSE	5′ TTTCTTCTGGCTGTGCCTTT 3′
SMN1 MUTANT REVERSE	5′ GGTAACGCCAGGGTTTTCC 3′
SMN2 WT FORWARD	5′ CTGACCTACCAGGGATGAGG 3′
SMN2 TRANSGENE	5′ GGTCTGTTCTACAGCCACAGC 3′
SMN2 WT REVERSE	5′ CCCAGGTGGTTTATAGACTCAGA 3′
SMNΔ7 TRANSGENE 01	5′ TCCATTTCCTTCTGGACCAC 3′
SMNΔ7 TRANSGENE 02	5′ ACCCATTCCACTTCCTTTTT 3′
SMNΔ7 POSITIVE CTRL FORWARD	5′ CAAATGTTGCTTGTCTGGTG 3′
SMNΔ7 POSITIVE CTRL REVERSE	5′ GTCAGTCGAGTGCACAGTTT 3′

### *In vivo* tests

Survival and other selected phenotypic tests to quantify key features and drug effects in the neonatal SMNΔ7 mouse model were evaluated. Kaplan–Meier survival curves were used to evaluate the survival of SMA + ERGO mice in comparison with SMA mice.

#### The hind limb suspension test

The hind limb suspension test or the tube test is a noninvasive motor function assessment specifically created for neonatal rodents. This test evaluates the proximal hind limb muscle weakness in mouse or rat neonates. The mouse is suspended upside down by its hind limbs in a centrifuge tube with a cotton ball to safeguard the animal's head in the event of a fall. Hind limb score (HLS) assesses the position of the legs and the tail [34]. The scoring is based on the following criteria: score 4, hind limb separation with elevated tail; score 3, limbs positioned closely together but rarely touching each other; score 2, hind limbs near each other, often in contact; score 1, hind limbs frequently in a clasped position with the tail raised [34].

#### Righting reflex

Pups are positioned on their back on a level surface, and their ability to move to a dorsal position was evaluated, recording the duration of time it takes until they return to the prone position [35].

#### Open arena test

The animals were placed in a 63 × 42 cm arena for 5 min, in which their performances were recorded by a SMART video tracking system (2 Biological Instruments; Besozzo, Varese, Italy) and a Sony CCD color video camera (PAL). Using this software, the total distance covered by the mice in centimeters was measurable [36].

#### Calculation of scores

Scores ranging from 1 to 4 were assigned to all measured parameters in 11‐day‐old pups (weight, hind limb suspension test, righting reflex, and total distance in open arena) (Table 2). To determine the changes in hair, only two scores were assigned: value of 1 = completely bald and 2 = some evidence of hair growth. All the individual scores were then added up, and the total value indicated the Aggregate Phenotypic Score (APS) of the mouse. The overall score ranges from 5 (minimum) to 18 (maximum).

**Table 2:** *In vivo* tests and relative scores.

Hind limb suspension test	Score 1	Score 2	Score 3	Score 4
	Very poor performance	Poor performance	Intermediate performance	Intermediate performance
Weight	0–3 g	3.01–4.34 g	4.35–5.68 g	> 5.69 g
Total distance	0–10 cm	10.1–40 cm	40.1–100 cm	> 100 cm
Righting reflex	> 15 s	5.1–15 s	3–5 s	0–2.9 s
Hair growth	No hair	Hair	\	\

### Quantitation of ERGO

The quantitation of ERGO was performed on samples of protein extract from SMA and SMA + ERGO diaphragms of young offspring by HILIC‐ESI‐HRMS.

#### Preparation of standard solution and calibration curve

A stock solution of L‐ergothioneine (L‐ERGO) was prepared by dissolving 2.5 mg of L‐ERGO in 10 mL of a solvent mixture containing 80% water and 20% acetonitrile (ACN), yielding a final concentration of 250 μg·mL^−1^. Fresh working standard solutions were subsequently prepared by serial dilution of the stock solution with a blank solution to achieve the following concentrations: 50.00; 25.00; 12.50; 6.25; 3.13; 1.56; 0.78; 0.39; 0.19; 0.09 and 0.05 ng·μL^−1^. Analyses were carried out in triplicate.

#### Samples preparation

Samples preparation procedure was adapted from [37]. Tissue samples of SMA and SMA + ERGO animals (containing 20 and 40 μg tissue, respectively) were mixed with 500 μL of ice‐cold methanol and vortexed for at least 30 s. The samples were then incubated at −4 °C for 30 min. Following incubation, the samples were centrifuged, and the resulting supernatants were evaporated to dryness under a gentle stream of nitrogen. The dried residues were reconstituted in a solvent mixture containing 80% water and 20% ACN, and any remaining debris was removed by a second centrifugation. The clarified supernatants were then transferred to glass inserts for LC–MS/MS analysis.

#### Chromatography and mass spectrometry conditions

Samples were analyzed using an AdvanceBio Glycan Mapping HILIC column (2.1 × 150 mm, 2.1 μm, 300 Å; Agilent Technologies, Santa Clara, CA, USA) on a Dionex UltiMate 3000 HPLC system (Thermo Scientific, San Jose, CA, USA). The mobile phases consisted of 0.1% formic acid (FA) in Milli‐Q water (mobile phase A) and 0.1% FA in 100% acetonitrile (mobile phase B). Chromatographic separation was achieved using the following gradient: an initial hold at 80% B for 2.0 min for sample loading, a linear decrease from 80 to 60% B over 10 min, followed by a reduction to 40% B over 3.0 min for column washing, and then re‐equilibration at 80% B for 10 min. The flow rate was maintained at 0.4 mL·min^−1^, and the column temperature was set to 35 °C. For mass spectrometric detection, the HILIC column was coupled to a Q Exactive Focus Orbitrap mass spectrometer (Thermo Scientific). The MS parameters were set as follows: sheath gas flow rate of 20 arbitrary units (AU), auxiliary gas flow rate of 5 AU, and an auxiliary gas temperature of 300 °C. Electrospray ionization (ESI) was operated at a spray voltage of 3.5 kV, with a capillary temperature of 275 °C and an S‐lens RF level of 50 V. Data acquisition was performed in the m/z range of 50–600 with a resolution of 70 000, using one microscan, an automatic gain control (AGC) target of 3 × 10^6^, and a maximum ion injection time of 100 ms. Instrument control and data acquisition were managed using xcalibur software (Thermo Scientific).

### OXYBLOT analysis

The preparation of the diaphragm samples and the procedure were described in the paper of Cadile *et al*. [6]. Protein oxidation was quantified by defining the oxidative index (OI), that is, the ratio between densitometric values of the OXYBLOT bands and those stained with Ponceau Red. The OI was expressed relative to control samples to compare different experiments.

### Western blot analysis

Diaphragm samples were processed as described by Cadile *et al*. [6]. The membranes were incubated with the specific primary antibody appropriately diluted in a solution of TBST 1X containing 5% BSA or 5% fat‐free milk depending on the specificities of the antibodies in the datasheet (Table 3). The target protein levels were normalized for the amount of a housekeeping protein (tubulin); the phosphorylation levels of some proteins were evaluated by the ratio between phosphorylated and unphosphorylated total forms of the same protein.

**Table 3:** Antibodies used for western blot analysis.

Primary antibody	Species	Dilution	Supplier	Catalog number
p‐AMPK	Rabbit	1 : 1000	Cell Signaling	#4188
AMPK	Rabbit	1 : 1000	Cell Signaling	#2532
PGC1α	Rabbit	1 : 1000	Abcam	Ab54481
TOM20	Rabbit	1 : 1000	Santa Cruz	sc‐11 415
DRP1 pSer637	Rabbit	1 : 1000	Cell Signaling	#4867
SOD1	Rabbit	1 : 1000	Abcam	Ab16831
Catalase	Rabbit	1 : 1000	Abcam	Ab52477
PRDX3	Rabbit	1 : 1000	Abcam	Ab73349
LC3B	Rabbit	1 : 1000	Sigma	L7543
p62	Rabbit	1 : 2000	Cell Signaling	#5114
PARKIN	Mouse	1 : 2000	Invitrogen	#39–0900
PINK1	Mouse	1 : 1000	Abcam	Ab75487
GPX	Mouse	1 : 1000	Abcam.	Ab22604
HMOX1	Mouse	1 : 1000	Santa Cruz	sc‐390 991
NRF2	Rabbit	1 : 1000	Abcam	Ab31163
KEAP1	Rabbit	1 : 1000	Cell Signaling	#4617
Tubulin	Mouse	1 : 2000	Sigma	T6199

### Electron microscopy (EM)

Samples for EM were prepared as described in Boncompagni *et al*. [37].

Quantitative analysis (Table 4). Columns A–C: incidence of the Ca2+ release units (CRUs), mitochondria, and CRUs‐mitochondriaairs, respectively, was evaluated in longitudinal sections and reported as average number over 100 μm ^2^ of area. Column D: the average size of mitochondria was quantified in longitudinal sectioned images by manually delineating the organelle contours using the jeol sightx‐viewer software (version 2.1.26.1818). All these parameters were evaluated in micrographs taken within the intermyofibrillar region of the fiber, whereas the subsarcolemmar regions were intentionally excluded to avoid, including clusters of mitochondria in the quantitation.

**Table 4:** Quantitative electron microscopy (EM) analysis of mitochondria and Ca2+ release units (CRUs) in WT, SMA, and SMA + ERGO. Data are shown as Mean ± SD. Columns A–D: data were obtained from longitudinal sections (as in images of Fig. 6); Columns E and F: data were obtained from cross‐sections (as in images of Fig. 7). (A) Anova *P < 0.0001 WT vs. SMA; ^ǂ^ *P* < 0.0001 SMA vs. ERGO. (B) Anova *P < 0.0004 WT vs. SMA and WT vs. ERGO. (C) Anova *P < 0.0001 WT vs. SMA and WT vs. ERGO; ^ǂ^ *P* < 0.0001 SMA vs. ERGO. (D) Anova *P* < 0.0017 WT vs. SMA; ^ǂ^ *P* < 0.0017 SMA vs. ERGO. (E) Anova *P < 0.0006 WT vs. SMA; ^ǂ^ *P* < 0.0001 SMA vs. ERGO. (F) Anova *P < 0.0004 WT vs. SM; ^ǂ^ *P* < 0.0001 SMA vs. ERGO. (A–C) Sample size: WT, *n* = 3 mice, 21 fibers, 3 micrographs/fiber; SMA, *n* = 2 mice, 10 fibers, 3 micrographs/fiber; ERGO, *n* = 2 mice, 11 fibers, 3 micrographs/fiber. (D) Sample size: WT, *n* = 2 mice, 5 fibers; SMA, *n* = 2 mice, 5 fibers; ERGO, *n* = 2 mice, 5 fibers. In parenthesis: total number of mitochondria analyzed. (E, F) Sample size: WT, *n* = 3 mice, 31 fibers, 1 micrograph/fiber; SMA, *n* = 2 mice, 51 fibers, 1 micrograph/fiber; ERGO, *n* = 2 mice, 22 fibers, 1 micrograph/fiber.

	A	B	C	D	E	F
	No. of Mitochondria/100 μm ^2^	No. of CRUs/100 μm ^2^	No. of CRU/mito pairs/100 μm ^2^	Average mitochondria size (μm ^2^)	Mitochondria volume/total volume (%)	Average no. of mitochondria undergoing mitophagy/100 μm ^2^
WT	58.5 ± 1.9	35.0 ± 1.2	19.5 ± 1.0	0.51 ± 0.03 (72)	23.4 ± 0.6	1.2 ± 0.3
SMA	37.3 ± 2.7*	26.5 ± 2.1*	9.8 ± 1.0*	0.63 ± 0.03 (103)	27.8 ± 1.2*	0.7 ± 0.2*
SMA+ ERGO	55.9 ± 2.5^ǂ^	28.2 ± 1.9*	14.9 ± 1.4*^ǂ^	0.50 ± 0.03^ǂ^ (125)	20.6 ± 3.0^ǂ^	2.7 ± 0.5^ǂ^

Columns E, F: mitochondrial volume (%) and incidence of mitochondria undergoing mitophagy reported as an average per 100 μm ^2^ were evaluated in cross‐sections. Mitochondrial volume was determined using the well‐established stereology point‐counting technique [38].

### Statistical analysis

The sample size was chosen to use the fewest number of animals to achieve statistical significance. By power calculation with the stata 13 and gpower 3.1 software, a sample size of 10 mice was estimated for each line with and without supplementation, but we had many difficulties in creating a stable and productive colony, so sometimes we were forced to use a lower number of animals. Quantitative variables were expressed as mean ± standard deviation (SD), while qualitative variables were presented as frequencies or percentages. Shapiro–Wilk's test was employed to assess normality of data, and Bartlett's test was used to test homogeneity of variance. For comparisons between two groups, Student's *t*‐test for independent data was applied. In cases of violations of the assumption of normality, the Mann–Whitney *U*‐test was used. For comparisons among more than two groups, one‐way ANOVA was employed. If assumptions for ANOVA were not met, the Kruskal–Wallis test was utilized. Kaplan–Meier analysis was conducted to compare survival curves. Statistical significance was determined at the 0.05 level (*P* < 0.05) in the two tails. Data analysis was performed using graphpad prism 9.0 and Stata® v17.

## Results

### Effect of ERGO *in vivo*

#### Ergo increased SMA mice survival

**Fig. 1:**
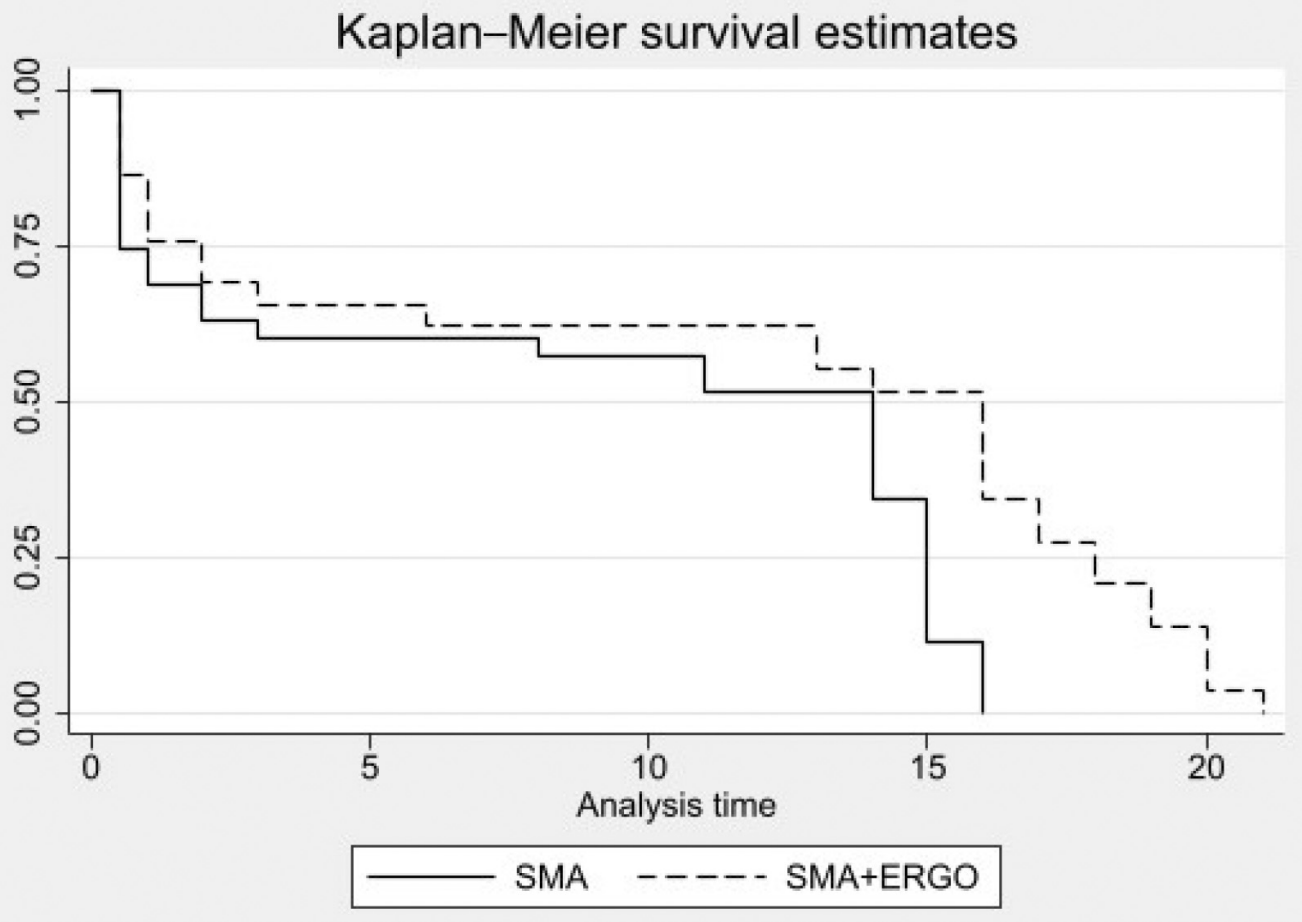
Kaplan–Meier survival curve. The graph shows the number of days on the *x*‐axis and the survival probability on the *y*‐axis. Solid trace SMA, *n* = 35; dotted trace SMA + ERGO, *n* = 29. Kaplan–Meier analysis was conducted to compare survival curves. The mean survival (+/− SD) of SMA and SMA + ERGO was 8.90 ± 1.13 and 11.28 ± 1.45, respectively. The log‐rank test was statistically significant (*P* = 0.002). ERGO, ergothioneine; SMA, spinal muscular atrophy.

According to the literature, SMA∆7 mice have an average lifespan of approximately 13 days [6, 38] This mouse model is a triple mutant that contains a knockout of mouse Smn1 (caused by insertion of a lacZ reporter) and two transgenes, one with a single normal copy of human SMN2 and the second with a human SMN2 promoter and a human SMN2 cDNA that lacks exon 7. The treatment with ERGO was found able to extend their survival to up to 21 days (Fig. 1). The difference in the overall survival was statistically significant (*P* = 0.002) demonstrating the possible efficacy of treatment with ERGO in increasing the survival probability of SMA mice.

#### Ergo improved the motor performance of SMA mice

**Fig. 2:**
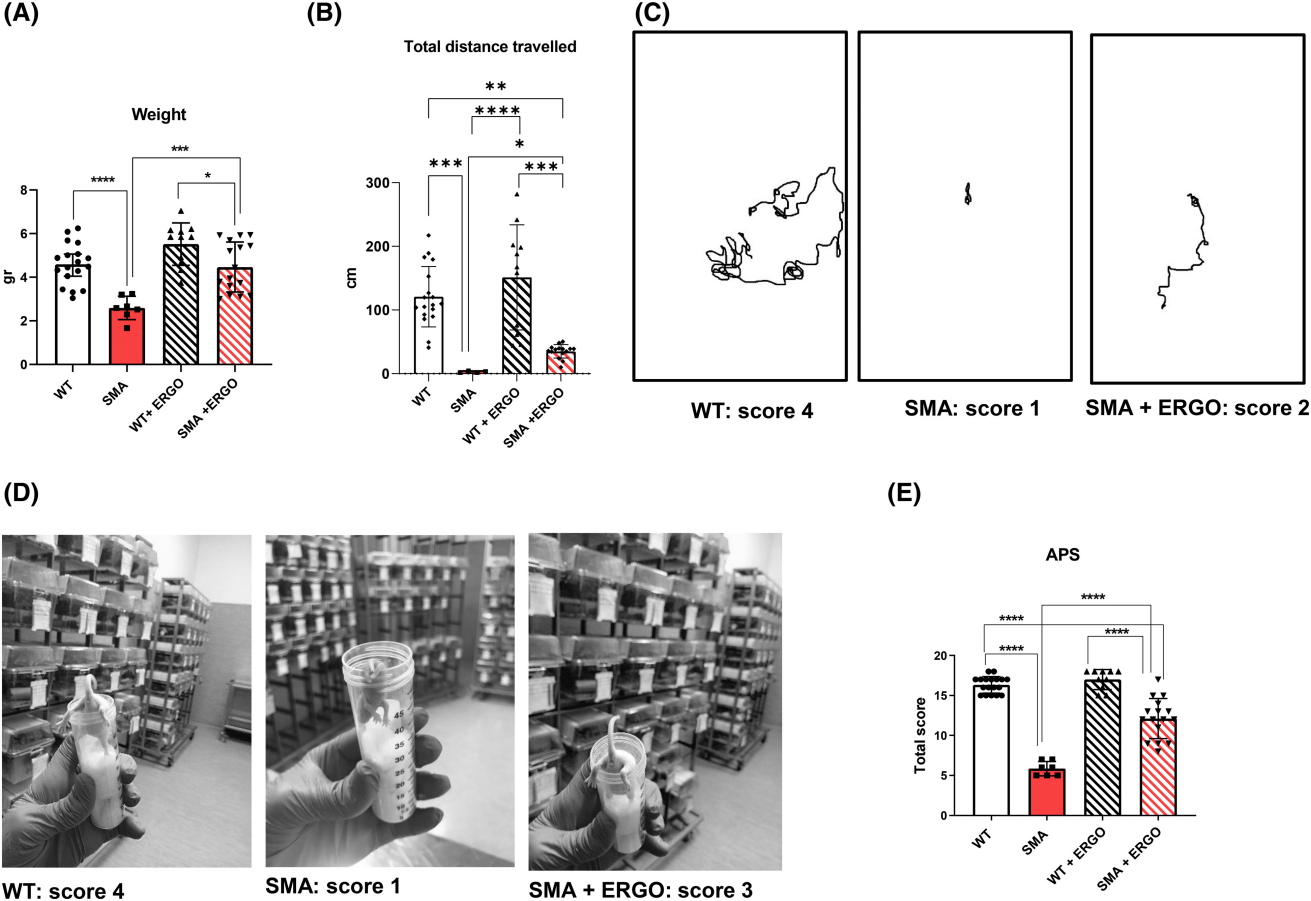
Parameters of health and motor performance. (A) weight of 11‐day‐old mice (WT, *n* = 18; SMA, *n* = 7; WT + ERGO, *n* = 9; SMA + ERGO, *n* = 16) (B) total distance traveled by 11‐day‐old mice (WT, *n* = 18; SMA, *n* = 7; WT + ERGO, *n* = 9; SMA + ERGO, *n* = 16) (C) maps pathway covered in an open arena of a WT, SMA, and a SMA + ERGO 11‐day‐old mouse, (D) representative hind limb suspension test of a WT, SMA, and SMA + ERGO 11‐day‐old mouse, (E) Aggregate Phenotype Score (APS) graph of 11‐day‐old mice. The graph includes the *in vivo* measured parameters: weight, hind limb suspension test, righting reflex, hair growth, and total distance covered in an open arena (WT, *n* = 18; SMA, *n* = 7; WT + ERGO, *n* = 9; SMA + ERGO, *n* = 16). For comparisons between two groups, Student's *t*‐test for independent data was applied. Bars represent means ± SD. Individual data are represented as scatter plots. *P ≤ 0.05; **P ≤ 0.01; ***P ≤ 0.001; ****P ≤ 0.0001. ERGO, ergothioneine; SMA, spinal muscular atrophy; WT, wild‐type.

ERGO significantly improved the motor performance of 11‐day‐old SMA mice, while not affecting WT mice (not shown). SMA + ERGO mice grew and developed much faster than the SMA (Fig. 2A). Regarding motor performance, SMA + ERGO mice exhibited higher motor activity (Fig. 2B,C) and lower muscle weakness (Fig. 2D), compared with the untreated SMA mice. All the parameters measured were combined in the total APS value (Fig. 2E). The differences among the groups were statistically significant (*P* < 0.001), showing that the SMA + ERGO mice had a better phenotype than the SMA mice. A significant difference remains between WT and SMA + ERGO mice.

### Quantitative determination of ERGO

Accurate ERGO quantitation was carried out by HILIC‐ESI‐HRMS applying the method previously reported. The ion signal at 230 m/z was extracted to selectively monitor ERGO in the total ion current trace. A calibration curve was built with standard L(−)ERGO in the range 5000–0.05 ng·mL^−1^, resulting in the equation *y* = 2.43*107 × + 5.31*106 (*R* ^2^ 0.999). Analyses were carried out in triplicate, with a mean RSD of 3.93%, indicating the reproducibility of the data and the robustness of the analytical procedure.

### Quantitation of ERGO in tissue

Quantitation of ERGO was carried out in four independent samples of protein extract from SMA and SMA + ERGO diaphragms by HILIC‐ESI‐HRMS as previously reported. The amount of ERGO in the samples was found to be 16.57 and 14.87 ng in SMA1 and SMA2, and 10.82 and 11.51 ng in SMA + ERGO1 and SMA + ERGO2, respectively (see Table S1). These data suggest only slight and nonsignificant differences in ERGO levels in the analyzed tissues. Notably, at the same retention time of ERGO, a second ion was detected with a 186 m/z. Based on the MW and retention, we hypothesized this molecule might be a decarboxylated metabolite of ERGO. Despite an absolute quantitative determination could not be carried out, it could be estimated by the relative abundance an approximately 2,5‐fold increase in SMA + ERGO samples in respect to SMA samples of this molecule. However, to support this hypothesis, additional experiments can be carried out in future studies.

### Effect of ERGO at molecular level

In our previous paper, we found the presence of altered mitochondria and impairment in the mitophagy process in SMA diaphragm [6]. Very recently, it was demonstrated that ERGO can enter mitochondria, suggesting a possible role in maintaining redox homeostasis and counteracting mitochondrial dysfunction in neurodegenerative diseases [21].

The effect of ERGO on the expression of markers linked to mitochondrial homeostasis in diaphragm muscle isolated from mice at the eleventh postnatal day of life was investigated.

#### ERGO reduced the expression of the markers of mitochondrial mass

**Fig. 3:**
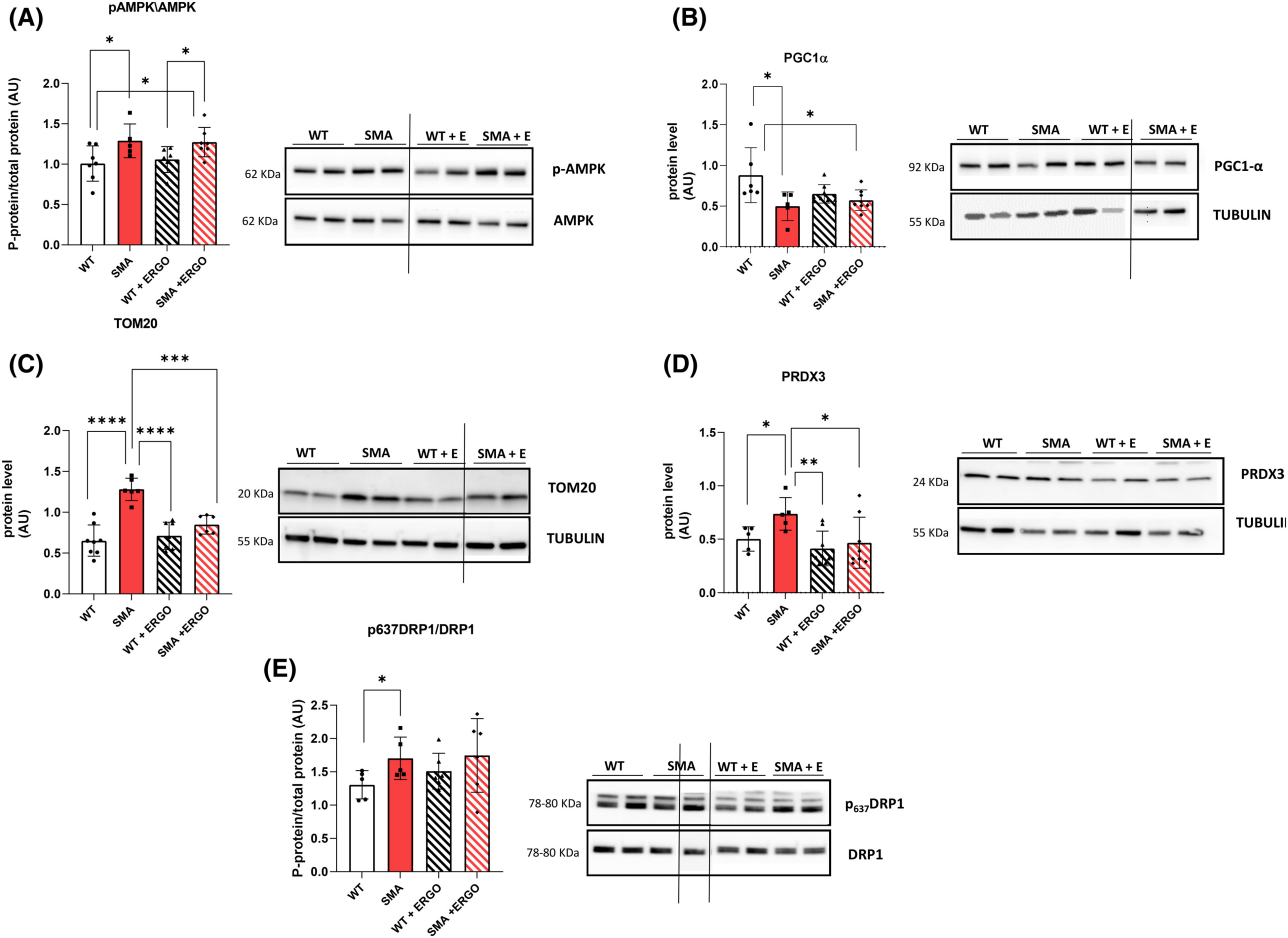
Effect of ERGO on energy balance, mitochondrial mass, and dynamics in diaphragm muscle. (A) Mean values of the ratio between the content in the phosphorylated (p) and total forms for AMPK determined by western blots (WT, *n* = 7; SMA, *n* = 5; WT+ ERGO, *n* = 6; SMA+ ERGO, *n* = 7). (B) Protein expression of PGC1α (WT, *n* = 6; SMA, *n* = 5; WT+ ERGO, *n* = 8; SMA+ ERGO, *n* = 8) and (C) mitochondrial import receptor subunit TOM‐20 (WT, *n* = 8; SMA, *n* = 6; WT+ ERGO, *n* = 7; SMA+ ERGO, *n* = 6) and (D) Piroxiredoxin 3 (PRDX3) (WT, *n* = 5; SMA, *n* = 5; WT+ ERGO, *n* = 8; SMA+ ERGO, *n* = 8), determined by western blots. (E) Phosphorylation of serine 637 of DPR1 (WT, *n* = 5; SMA, *n* = 5; WT+ ERGO, *n* = 6; SMA+ ERGO, *n* = 6). The level of the protein target was normalized against the level of the housekeeping tubulin measured in the same blot. Representative western blots are shown. Dividing lines have been inserted to highlight the images formed by different parts of the same gel or from different gels. ERGO, ergothioneine; SMA, spinal muscular atrophy; WT, wild‐type. For comparisons between two groups, Student's *t*‐test for independent data was applied. Bars represent means ± SD. Individual data are represented as scatter plots. *P ≤ 0.05, **P ≤ 0.01, ***P ≤ 0.001, ****P ≤ 0.0001.

ERGO did not affect the phosphorylation of the energy sensor AMPK and the expression of the mitochondrial proteins in WT mice (Fig. 3). However, the AMPK phosphorylation was significantly increased in SMA diaphragm compared with WT, suggesting the presence of an energy imbalance, which was maintained in SMA + ERGO (Fig. 3A). ERGO did not affect the mitochondrial biogenesis regulator PGC1α whose expression was significantly lower in both SMA and SMA + ERGO in comparison with WT (Fig. 3B).

However, the expression of markers of mitochondrial mass (TOM‐20 and PRDX3), which was significantly increased in SMA, returned to WT levels after ERGO treatment, notwithstanding the small sample size (Fig. 3C,D).

The expression of specific proteins involved in mitochondrial dynamics was assessed and no significant difference following ERGO treatment was found, whereas a significant increase in the phosphorylation at serine 637 in DPR1, according to the paper of Cadile *et al*. [6], was found in SMA (Fig. 3E).

#### ERGO increased the expression of the markers of autophagy and mitophagy

**Fig. 4:**
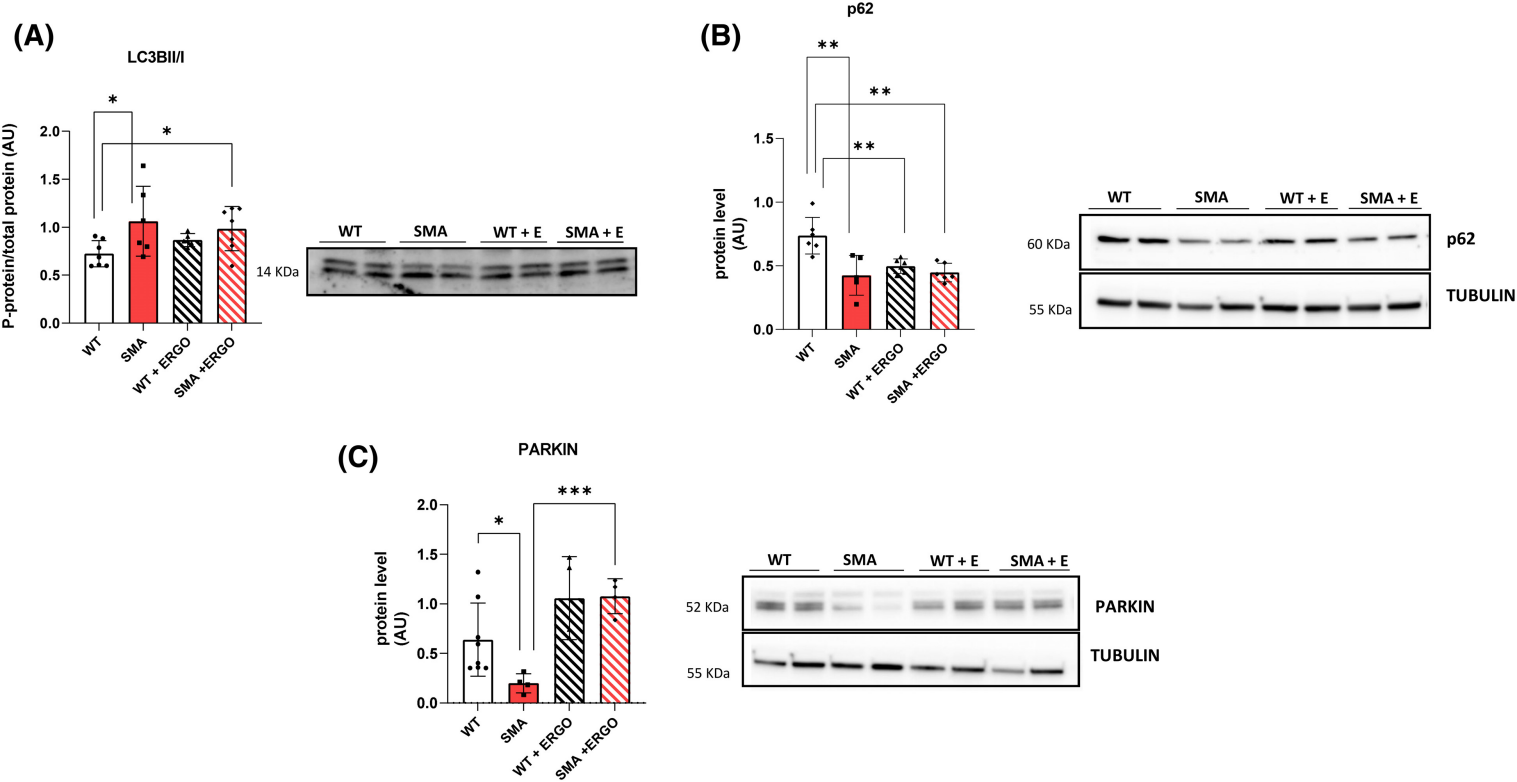
Effect of ERGO on autophagy and mitophagy in diaphragm muscle. (A) The ratio between the active form and the inactive form of LC3B (LC3BII/LC3BI) (WT, *n* = 7; SMA, *n* = 6; WT+ ERGO, *n* = 5; SMA+ ERGO, *n* = 7), and (B) protein levels of p62 (WT, *n* = 6; SMA, *n* = 5; WT+ ERGO, *n* = 6; SMA+ ERGO, *n* = 6), determined by western blots. (C) Protein levels of mitophagy maker PARKIN (WT, *n* = 8; SMA, *n* = 4; WT+ ERGO, *n* = 6; SMA+ ERGO, *n* = 6), determined by western blots. The level of the protein target was normalized against the level of the housekeeping tubulin measured in the same blot. Representative western blots are shown. For comparisons between two groups, Student's *t*‐test for independent data was applied. Bars represent means ± SD. Individual data are represented as scatter plots. *P ≤ 0.05 **P ≤ 0.01, ***P ≤ 0.001. ERGO, ergothioneine; SMA, spinal muscular atrophy; WT, wild‐type.

ERGO did not affect the lipidation of the autophagic marker LC3 (LC3B) but induced a significant reduction in the scaffold protein p62 in WT mice (Fig. 4A,B). In SMA diaphragm, according to the paper of Cadile *et al*., a significant increase in the active form LC3B along with a significant decrease in p62 expression in comparison with WT was found, suggesting the activation of the autophagy process (Fig. 4A,B) [6]. As we did not perform measurements of autophagic flux, it is difficult to determine how autophagy is altered. We did however take out tissues all at the same time of the day, therefore eliminating any changes due to circadian fluctuations of autophagy.

Finally, the expression of Parkin, a protein involved in promoting the selective autophagy of mitochondria, was not changed in WT but was significantly decreased in SMA and normalized in SMA + ERGO notwithstanding the small sample size, suggesting that ERGO was able to stimulate the mitophagy process only in SMA mice (Fig. 4C). This could potentially explain the reductions in mitochondrial markers like TOM20. No differences were found in the expression of PINK1 (Fig. S1).

#### ERGO seemed to reduce redox imbalance acting on the GPX antioxidant system

**Fig. 5:**
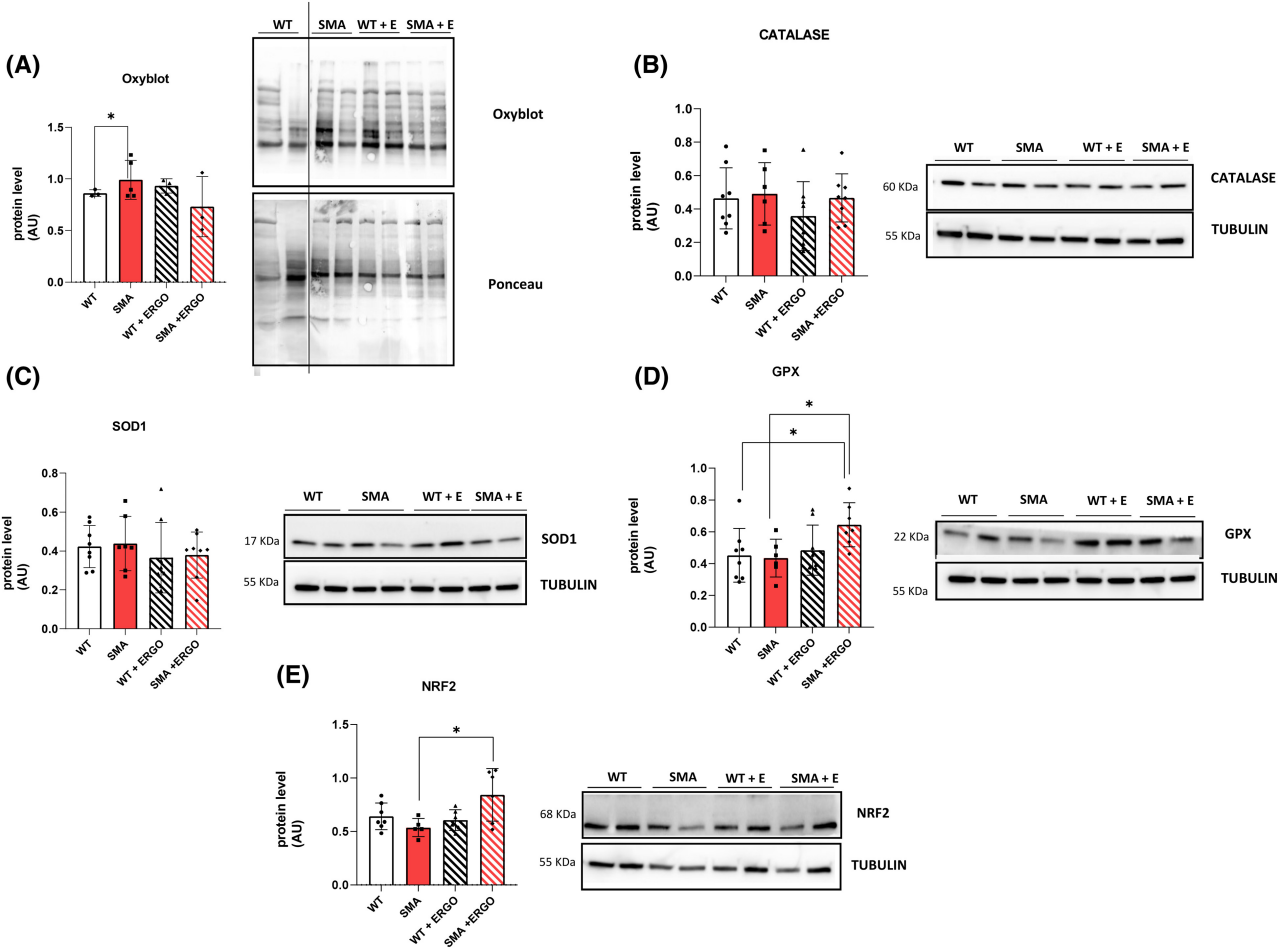
Effect of ERGO on redox imbalance in diaphragm muscle. (A) Protein levels of carbonylated proteins revealed by oxyblot analysis (WT, *n* = 3; SMA, *n* = 5; WT+ ERGO, *n* = 3; SMA+ ERGO, *n* = 3). Dividing line has been inserted to highlight the images formed by different parts from different gels. (B) Protein levels of catalase (WT, *n* = 8; SMA, *n* = 6; WT+ ERGO, *n* = 8; SMA+ ERGO, *n* = 8). (C) Protein levels of SOD1 (WT, *n* = 8; SMA, *n* = 7; WT+ ERGO, *n* = 8; SMA+ ERGO, *n* = 8). (D) Protein levels of GPX (WT, *n* = 8; SMA, *n* = 6; WT+ ERGO, *n* = 8; SMA+ ERGO, *n* = 7). (E) Protein levels of NRF2 (WT, *n* = 7; SMA, *n* = 5; WT+ ERGO, *n* = 6; SMA+ ERGO, *n* = 5) determined by western blots. The level of the protein target was normalized against the level of the housekeeping tubulin measured in the same blot. Representative western blots are shown. For comparisons between two groups, Student's *t*‐test for independent data was applied. Bars represent means ± SD. Individual data are represented as scatter plots. *P ≤ 0.05. ERGO, ergothioneine; SMA, spinal muscular atrophy; WT, wild‐type.

Oxyblot analysis showed the presence of a higher level of carbonylated protein in SMA in comparison with WT (Fig. 5A). ROS accumulation was most likely due to the lack of activation of the main antioxidant systems (SOD1, Catalase) (Fig. 5B,C).

ERGO treatment seemed to reduce the level of carbonylated proteins, but the difference between SMA and SMA + ERGO was not significant (Fig. 5A) as well as the protein level of the oxidative marker HMOX1 (Fig. S2). It should be noted that the data are based on a very small number of animals and therefore deserve to be confirmed.

The ERGO effect could be related to the stimulation of the GPX antioxidant system (Fig. 5D). The NRF2/KEAP1 pathways were also investigated. ERGO increased the expression of NRF2 (Fig. 5E) but not that of KEAP1 (Fig. S3).

### Effect of ERGO on mitochondria

A detailed (quantitative and qualitative) ultrastructural analysis was performed on diaphragm cross‐sections. The diaphragm is a highly oxidative skeletal muscle [39]. Oxidative fibers have a high density of mitochondria which may acquire different disposition/organization [40]. In adult oxidative fibers, the majority of mitochondria appear in longitudinal sections as oval/elongated profiles symmetrically positioned within the intermyofibrillar spaces on either side of the Z‐line adjacent to the CRU (triadic mitochondria). Triadic mitochondria appear often in continuous with a smaller subset of elongated mitochondria, assembled in quite long longitudinal columns between myofibrils (longitudinal mitochondria). Finally, a single layer of longitudinal subsarcolemmal mitochondria is also frequently visible in diaphragmatic oxidative fibers [41].

#### ERGO restored the incidence of mitochondria, Ca2+ release units (CRUs) and their reciprocal association

**Fig. 6:**
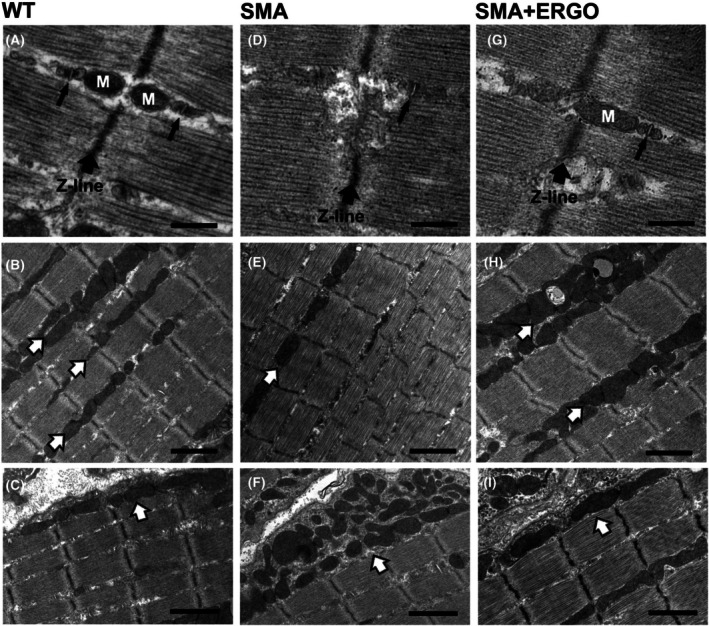
Effect of ERGO on mitochondria and Ca2+ release units (CRUs) distribution and their reciprocal association in diaphragm muscle. Representative electron microscopy (EM) images of longitudinal sections of diaphragm muscles from WT (A–C), SMA (D–F), and SMA + ERGO (G–I) mice at 11 days of age. Images in panels D–F, are representative of SMA diaphragms showing: (i) absence of structural association of a mitochondrion with a CRU (black small arrow; D), (ii) extensive fiber region with deficiency of longitudinal columns of mitochondria; note: only one is visible (white, black‐outline arrow; E); (iii) the appearance of an abnormal cluster of mitochondria under the sarcolemma (white, black‐outline arrow; F). Labeling: M as for mitochondrion (A and G); large black arrows point to Z‐line; small arrows point to CRUs (A, D and G); white, black‐outlined arrows point to columns of longitudinal mitochondria between myofibrils (B, E and H) and to subsarcolemmal clusters of mitochondria (C, F and I). Scale bars: 0.2 μm (A, D and G); 2 μm (B, C, E, F and H, I). SMA, spinal muscular atrophy; ERGO, ergothioneine; WT, wild‐type.

In WT diaphragmatic fibers from 11‐day‐old mice, *triadic* mitochondria are more rarely found due to fiber immaturity (Fig. 6A, black small arrows). On the contrary, at this age, mitochondria are frequently arranged in longitudinal columns between the myofibrils (Fig. 6B, white, black‐outlined arrows). A short, single layer of *longitudinal* mitochondria may be detected under the sarcolemma (Fig. 6C, white, black‐outlined arrow). However, in SMA diaphragms, mitochondria disposition appeared altered compared with the WT. Indeed, in about 60% of the fibers analyzed, we noted several areas where *triadic* mitochondria were apparently missing (Fig. 6D, black small arrow). Moreover, extensive regions with few or almost completely absent *longitudinal* mitochondria (Fig. 6E, white arrows) were often detected. Interestingly, in the same fibers, abnormal accruals of mitochondria under the sarcolemma were frequently observed (Fig. 6F, white, black‐outlined arrow) Finally, higher magnification allowed us to detect that in the free mitochondria regions, CRUs were also usually missing (not shown).

However, analysis of diaphragm fibers from mice treated with ERGO revealed an ultrastructural disposition/organization of mitochondria and CRUs apparently similar to that of WT diaphragms (Fig. 6G–I). A detailed quantitative analysis performed within longitudinally sectioned fibers and reported in Table 4 strongly supported the visual observation. We evaluated the incidence of mitochondria, CRUs, and mitochondria–CRU pairs (Table 4) within the fiber interior, that is, excluding the subsarcolemmal regions (see Material and Methods for details). Number/area of both mitochondria and CRUs is decreased in SMA compared with that in WT (Table 4, Columns A and B), which in turn caused a great reduction in the number/100 μm ^2^ of mitochondria–CRU pairs, that is, the number of mitochondria structurally associated with a CRU (Column C). Treatment with ERGO was able to significantly rescue the number/100 μm ^2^ of mitochondria and of mitochondria–CRU pairs (Table 4, Columns A and C) although it was not able to significantly rescue the number/100 μm ^2^ of CRUs (Table 4, Column B).

#### ERGO restored mitochondria volume and increased incidence of mitochondria undergoing mitophagy

**Fig. 7:**
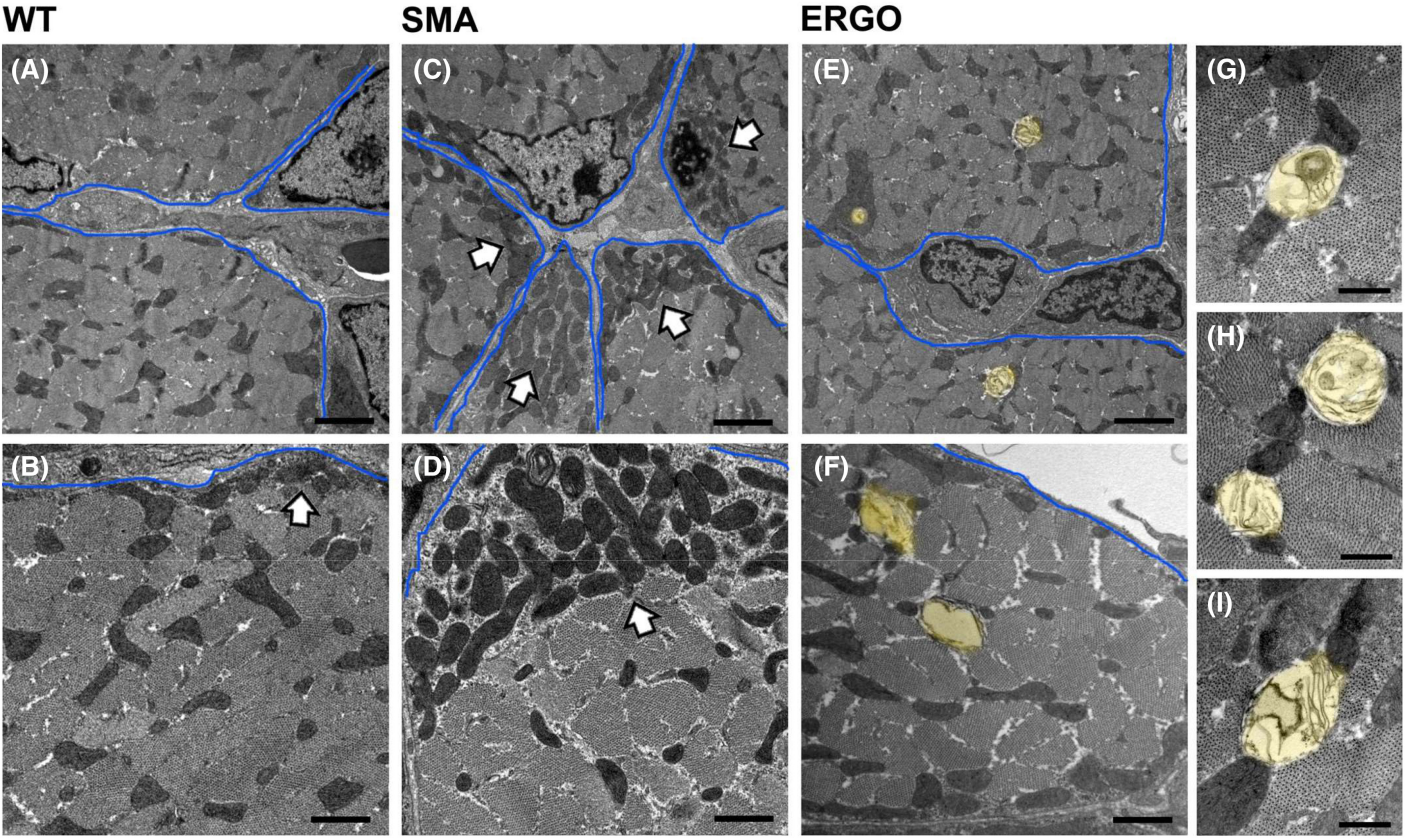
Effect of ERGO on mitochondria volume and mitochondria mitophagy in diaphragm muscle. Abnormal accrual of mitochondria is very rare under the sarcolemma of WT fibers (A and B, white, black‐outlined arrow). Subsarcolemmal clusters of mitochondria are frequently present in SMA diaphragms (C and D, white black‐outline arrows). In SMA + ERGO diaphragms mitochondria undergoing mitophagy appeared frequently (E–I). Labeling: white, black‐outlined arrows point to subsarcolemmal clusters of mitochondria; sarcolemma membranes were outlined in blue; mitochondria undergoing mitophagy were falsely labeled in light yellow for easier visualization. Scale bars: 2 μm (A, C and E); 1 μm (B, D and F); 0.5 μm (G, H and I). SMA, spinal muscular atrophy; ERGO, ergothioneine; WT, wild‐type.

In WT diaphragms at 11 days, subsarcolemmal mitochondria were not very frequent and usually arranged in a short, single layer when they were present (Figs 6C and 7A,B, white arrows). In SMA diaphragms, the presence of fiber regions lacking intermyofibrillar mitochondria was often associated with the appearance of abnormal subsarcolemmal clusters of mitochondria, suggesting a translocation and/or redistribution of mitochondria from their triadic or longitudinal position within the intermyofibrillar spaces to the subsarcolemmal region (Figs 6 and 7C,D, white arrows). Finally, we did not notice a particularly high presence of morphologically altered mitochondria in any of the different samples analyzed. However, in SMA + ERGO fibers, we detected a higher presence of mitochondria undergoing mitophagy compared with diaphragms from WT and SMA mice (Fig. 7E–I).

In cross‐sectioned fibers from WT, SMA, and SMA + ERGO fibers, we quantify the total mitochondria volume (subsarcolemmal mitochondria included; see Materials and Methods for details) and the incidence of mitochondria undergoing mitophagy (Table 4, columns D and E). The relative fiber volume occupied by mitochondria (expressed as %/total fiber volume) was significantly higher in SMA diaphragms than in both WT and SMA + ERGO. Interestingly, in SMA + ERGO diaphragms, we detected a higher number of mitochondria undergoing mitophagy compared with both WT and SMA muscles (Fig. 7E–I and Table 4, Column E).

## Discussion

The present study builds upon our previous findings, which demonstrated that the acute local perfusion with a very powerful natural antioxidant molecule, ERGO, was able to significantly reduce SMA diaphragm fatigability [6]. Therefore, the results obtained in the paper of Cadile *et al*. support the rationale to test the effect of a dietary supplementation with ERGO in SMNΔ7 mice. ERGO is an antioxidant molecule that acts as a powerful scavenger of mitochondria‐derived superoxide species and a protector of cell constituents from damage by ROS [30, 42]. ERGO is also a histidine derivative that contains an imidazole ring suitable to function as a pH buffer [39]. Histidine compounds, such as carnosine, act as powerful buffers and attenuate changes in intracellular pH in muscles during anaerobic exercise [40]. Based on this information, it is possible to assume that ERGO was able to bind H^+^ ions derived from lactic acid that has accumulated following repeated high‐frequency contractions of the isolated diaphragm. The accumulation of lactic acid in muscle has historically been suggested to be the major cause of muscle fatigue [41]. Beyond that, ERGO could protect the diaphragm from excessive oxidation by stimulating mitochondrial function and reducing ROS formation, delaying the onset of fatigue. The significant increase in fatigue resistance following ERGO exposure could delay respiratory failure in SMA mice, increasing their survival. Moreover, increasing the resistance to fatigue of breathing muscles, ERGO could also increase the limb blood flow through a metabolic reflex, reducing locomotor muscle fatigue [43].

In the present study, ERGO was administrated at a dose of 20 μg/day to females in drinking water during pregnancy and while breastfeeding. The selected dose of 20 μg/day of ergothioneine was based on previous *in vivo* studies in mice demonstrating safety and beneficial effects on neuromuscular function [24, 25].

Herein, firstly, we demonstrated that ERGO treatment significantly extends the lifespan and enhances locomotor skills of SMA mice. Very importantly, mice treated with ERGO managed to survive 30% longer than their untreated SMA siblings. Accordingly, it was recently shown that ERGO was able to extend the lifespan of Drosophila [44] and aged mice [45] through, respectively, autophagy stimulation and radical‐removal activity.

ERGO was able to increase the SMA pup's growth and motor performance at 11th postnatal day of life but had no impact on WT mice. Indeed, ERGO is thought to be an adaptive antioxidant, that is, its antioxidant properties come into play mainly when the tissues are damaged [32].

The observed ERGO effect *in vivo* could be related to its ability to ameliorate diaphragm's functionality. To answer this question, the diaphragm of WT, SMA, WT + ERGO, and SMA + ERGO mice was analyzed at the molecular level.

The quantitation of ERGO was performed on samples of protein extract from SMA and SMA + ERGO diaphragms of young offspring by HILIC‐ESI‐HRMS and resulted in nonsignificant variations in ERGO (230 m/z) content among the samples. We speculate that mice, both supplemented and nonsupplemented, get the same amount of ERGO from the diet. However, we found a 2,5‐fold increase of an ERGO metabolite in supplemented animals.

At first, the effect of ERGO on WT diaphragm was assessed. No effect of ERGO in WT diaphragm was found except for the stimulation of the autophagic process, which could, however, contribute to the long‐term health of the mice [46].

The energy balance defect found in SMA diaphragm persisted in SMA + ERGO. We have previously hypothesized [6] that the energy imbalance in SMA was due to the presence of a number of dysfunctional mitochondria that could account for the increase in mitochondrial proteins TOM‐20 and PRDX3 but not contribute to an increase in mitochondrial activity. The decreased expression of the mitochondrial proteins in SMA + ERGO could be due to a reduction in the number of dysfunctional mitochondria. The remaining ones are probably too few (note that ERGO does not stimulate PGC1α) to solve the energetic imbalance.

In SMA + ERGO, a significant increase in Parkin protein expression was found, suggesting the activation of the mitophagic process by ERGO. In support of this hypothesis, E EM analysis showed that the number of intermyofibrillar mitochondria, CRUs, and mitochondria/CRUs pairs, that is, the number of mitochondria structurally associated with a CRU, were significantly reduced in SMA fibers, as the total mitochondrial volume and all these parameters were significantly rescued after treatment with ERGO. Moreover, EM analysis showed a significant increase in mitochondria undergoing mitophagy in SMA + ERGO mice.

Mitophagy plays a crucial role in maintaining cellular homeostasis, especially in high‐energy‐demand tissues, such as skeletal muscle [47]. Importantly, both molecular and EM data indicated that ERGO stimulates this process, which could help maintain mitochondrial turnover by eliminating the aberrant mitochondria. It was demonstrated that ERGO might protect mitochondrial DNA from ROS [48] and selective damage to mitochondrial DNA by oxidative stress has been implicated in SMA [49, 50]. However, if this was the only mode of action of ERGO, there would be no need to activate mitophagy. Since we found signals of mitophagy activation, the most likely explanation is that both pathways may occur.

Given the importance of mitochondria in the regulation of oxidative stress, this effect of ERGO could be crucial in countering the progressive muscle weakness and respiratory failure observed in SMA, which is a major cause of mortality in SMA mice, and probably also in human patients. ERGO was found to increase NRF2 and GPX expression, with no impact on other antioxidant enzymes, such as SOD1 and catalase. The main biological role of GPX is to protect the organism from oxidative damage [51]. The enhancement of GPX expression is likely to reduce the burden of oxidative stress in the SMA diaphragm. Previous studies have highlighted the contribution of oxidative stress to disease progression in SMA [1]. However, while we expected to observe a decrease in ROS accumulation in SMA+ ERGO mice, OXYBLOT analysis showed only a decreasing trend in carbonylated protein amounts in SMA+ ERGO mice in comparison with SMA. This could suggest that only mild reductions in oxidative stress are sufficient to ameliorate the disease phenotype.

Based on the obtained results, it is possible to state that ERGO is a very powerful and promising antioxidant compound able to promote longevity and motor manifestations in SMNΔ7 mice. However, it is not possible to say that *in vivo* ERGO effects can be only due to the antioxidant action on the diaphragm, but other targets must be considered. Similarly, the observed increase in survival is probably due to ERGO's broader effects across multiple physiological systems. Mitochondrial disturbances and ROS accumulation were found also in locomotor muscles of many mouse models of SMA [1, 5] and consequentially, we could suggest that the antioxidant effect of ERGO could ameliorate the locomotor capacity of the pups, that, in turn, could have better access to breast milk and consequently improve their life's quality and survival.

Finally, an action on MNs at central and/or peripheral levels cannot be excluded.

## Conclusion

The results of the current study demonstrated that ERGO treatment has a very significant positive effect on SMNΔ7 mice, especially on survival and locomotor skills. At the molecular level, it has been observed that ERGO has the ability to stimulate mitophagy, resulting in the elimination of nonfunctional mitochondria in the diaphragm of SMNΔ7 mice. However, the increase in survival could be due to an overall effect on multiple physiological systems.

These findings are significant also in a translational perspective. While ERGO cannot replace the efficacy of newly available SMA therapies, it presents an appealing option as a complementary treatment. ERGO could be particularly beneficial for patients who do not fully respond to existing therapies, offering a potential means to enhance the quality of life. Moreover, ERGO's safety and compatibility for use during gestation present a notable advantage. This could provide a larger therapeutic window, potentially improving outcomes in early‐stage SMA intervention.

So, these promising findings open the possibility for further research into ERGO as a novel adjuvant therapy for SMA. Future studies should focus on exploring ERGO interactions with other approved SMA therapies and its potential application in human clinical trials.

## Author contributions

MC and PR contributed to the conceptualization and resources. FC, DR, GR, CT, SK, and SB contributed to the investigation. MC contributed to the writing—original draft preparation, project administration, and funding acquisition. DR, SB, and PR critically reviewed the paper. OEF contributed to the statistical analysis.

## Conflict of interest

The authors declare no conflict of interest.

## Supporting information


**Fig. S1.** Protein expression of PINK1.
**Fig. S2**. Protein expression of HMOX1.
**Fig. S3**. Protein expression of KEAP1.
**Table S1**. ERGO quantitation in muscles samples.

## Data Availability

The data that support the findings of this study are available from the corresponding author [canepari@unipv.it] upon reasonable request.
