# Supplementary material for: Ergothioneine supplementation improves pup phenotype and survival in a murine model of spinal muscular atrophy
Source: FEBS Lett. 2025 Aug 6;599(21):3086–102. doi: 10.1002/1873-3468.70136 (PMC12599606; doi:10.1002/1873-3468.70136)
Supplement: Supplementary file 1 — Fig. S1. Protein expression of PINK1. Fig. S2. Protein expression of HMOX1. Fig. S3. Protein expression of KEAP1. Table S1. ERGO quantitation in muscles samples. [file FEB2-599-3086-s001.pdf]

Supplementary Fig.S1

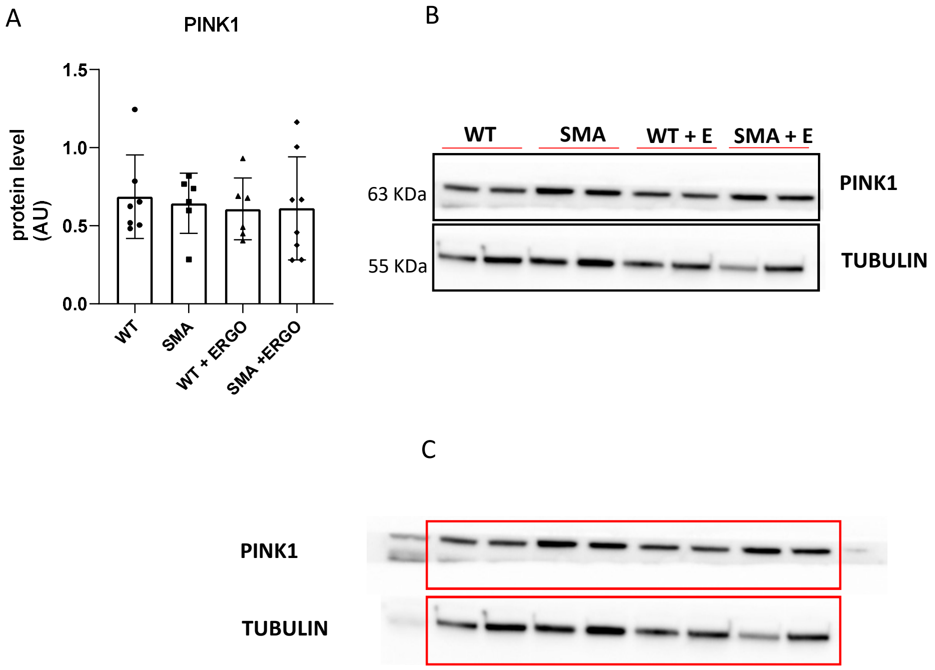

Protein expression of PINK1

(A) Protein expression of PINK1 (WT, n = 7 SMA, n = 6; WT+ ERGO, n= 6; SMA+ ERGO, n= 8). The level of the protein target was normalized against the level of the housekeeping tubulin measured in the same blot. Representative Western blots are shown (B). (C) Full blots

Supplementary Fig.S2

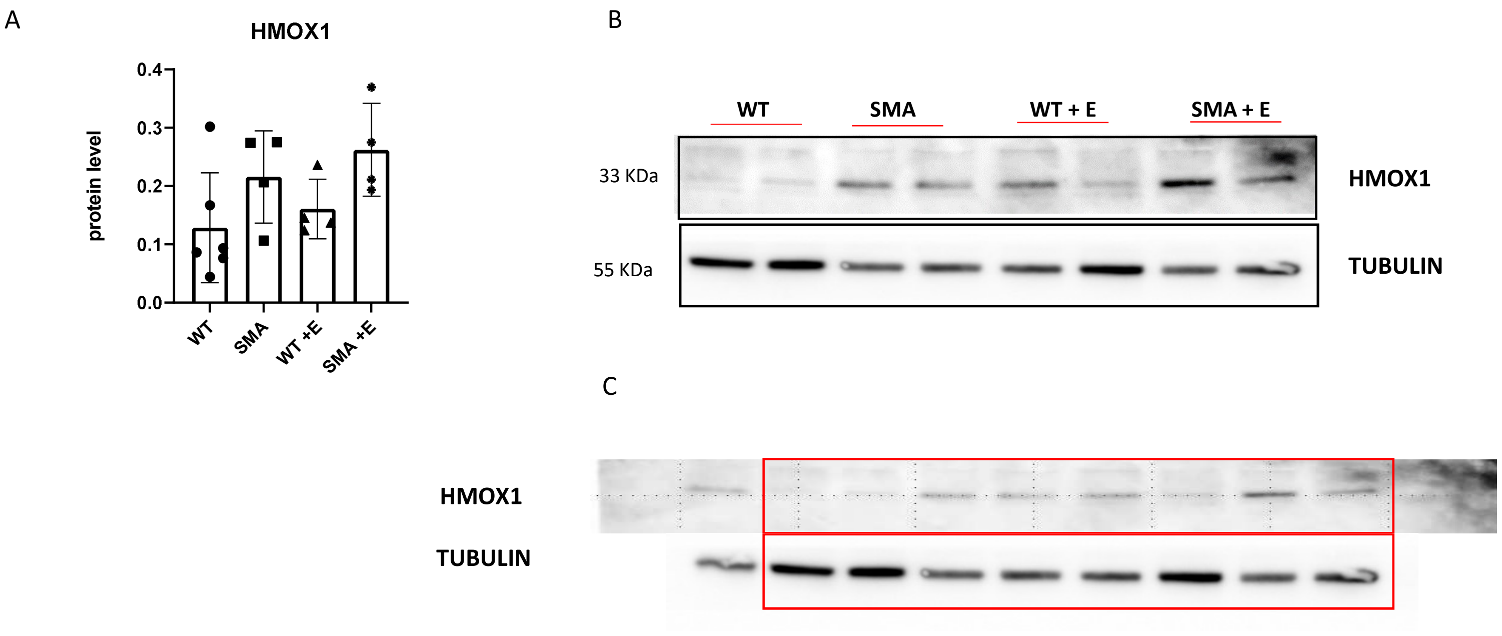

Protein expression of HMOX1

(A) Protein expression of HMOX1 (WT, n = 6 SMA, n = 4; WT+ ERGO, n= 4; SMA+ ERGO, n= 4). The level of the protein target was normalized against the level of the housekeeping tubulin measured in the same blot. Representative Western blots are shown (B). (C) Full blots

Supplementary Fig.S3

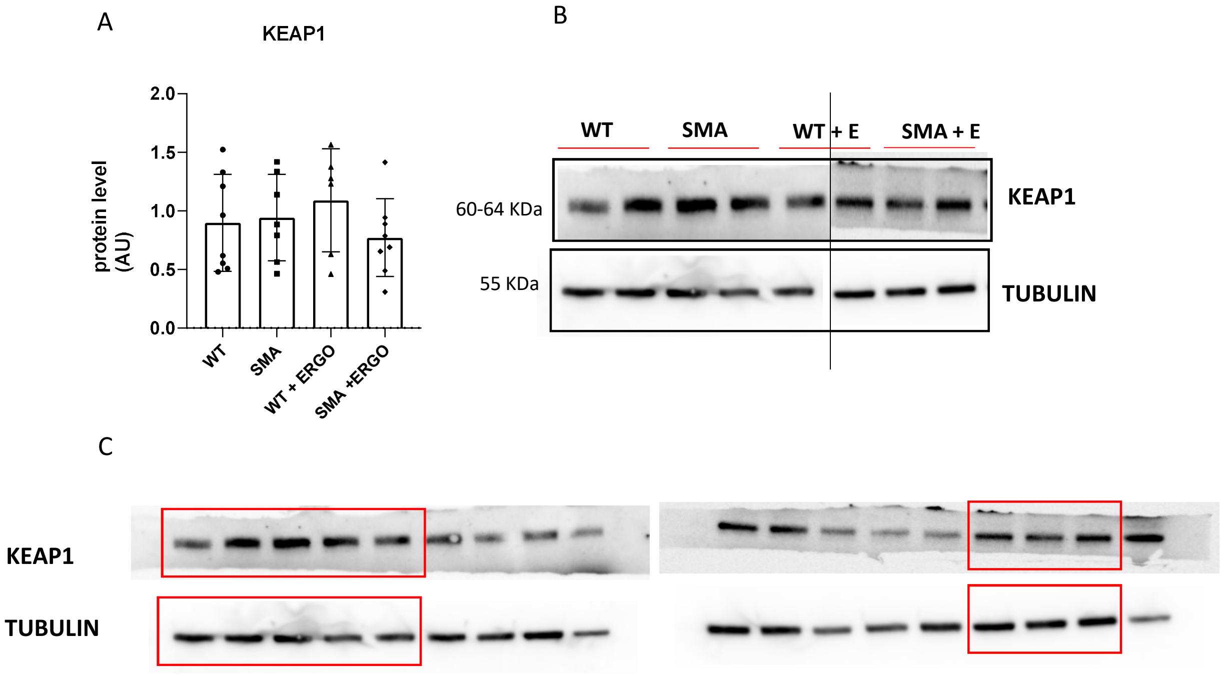

Protein expression of KEAP1

(A) Protein expression of KEAP1 (WT, n = 8 SMA, n = 7; WT+ ERGO, n= 6; SMA+ ERGO, n= 8). The level of the protein target was normalized against the level of the housekeeping tubulin measured in the same blot. Representative Western blots are shown (B) Dividing lines have been inserted to highlight the images formed by different parts of the same gel or from different gels (C) Full blots

Supplementary Table.S1

SAMPLE	ug ERGO total
SMA 10	16,57
SMA+ERGO 23	10,82
SMA 11	14,87
SMA+ERGO 24	11,51

ERGO quantitation in muscles samples
Amount of ERGO in four independent samples of protein extract from SMA and SMA+ERGO diaphragms measured by HILIC-ESI-HRMS
